# Density dependence of daily activity in three ungulate species

**DOI:** 10.1002/ece3.7570

**Published:** 2021-05-01

**Authors:** Juan Ignacio Ramirez, Joeri A. Zwerts, Marijke van Kuijk, Palma Iacobelli, Xuqing Li, Natalie Herdoiza, Patrick A. Jansen

**Affiliations:** ^1^ Department of Ecology and Environmental Sciences Umeå University Umeå Sweden; ^2^ Department of Environmental Sciences Wageningen University & Research Wageningen The Netherlands; ^3^ Colegio de Ciencias Biológicas y Ambientales Universidad San Francisco de Quito USFQ Quito Ecuador; ^4^ Institute of Environmental Biology Utrecht University Utrecht The Netherlands; ^5^ Copernicus Institute of Sustainable Development Utrecht University Utrecht The Netherlands; ^6^ Smithsonian Tropical Research Institute Panama Panama

**Keywords:** behavior, camera traps, food availability, hunting, landscape of fear, safety‐in‐numbers, temperate forest

## Abstract

Daily activity in herbivores reflects a balance between finding food and safety. The safety‐in‐numbers theory predicts that living in higher population densities increases safety, which should affect this balance. High‐density populations are thus expected to show a more even distribution of activity—that is, spread—and higher activity levels across the day. We tested these predictions for three ungulate species; red deer (*Cervus elaphus*), roe deer (*Capreolus capreolus*), and wild boar (*Sus scrofa*). We used camera traps to measure the level and spread of activity across ten forest sites at the Veluwe, the Netherlands, that widely range in ungulate density. Food availability and hunting levels were included as covariates. Daily activity was more evenly distributed when population density was higher for all three species. Both deer species showed relatively more feeding activity in broad daylight and wild boar during dusk. Activity level increased with population density only for wild boar. Food availability and hunting showed no correlation with activity patterns. These findings indicate that ungulate activity is to some degree density dependent. However, while these patterns might result from larger populations feeling safer as the safety‐in‐numbers theory states, we cannot rule out that they are the outcome of greater intraspecific competition for food, forcing animals to forage during suboptimal times of the day. Overall, this study demonstrates that wild ungulates adjust their activity spread and level based on their population size.

## Graphical abstract

Daily activity of three ungulate species is density dependent across ten temperate forest sites. Activity spread and level increases with ungulate population size. Suggesting that future research should account for population size as a driver of ungulate activity.

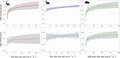

## 1 INTRODUCTION

Daily activity patterns—the distribution of activity throughout day and night—are a key feature of animal responses to their environment (Rowcliffe et al., 2014). Activity adaptations allow animals to optimize fitness in a changing environment by balancing the need to secure resources and the need to avoid predation (Bridges et al., 2004; Brook et al., 2012; Kronfeld‐Schor & Dayan, 2003; Levy et al., 2012). In addition, a variety of abiotic factors have been found to affect daily activity, including seasonality (Ikeda et al., 2016), day length (Vazquez et al., 2019), temperature (Bennie et al., 2014; McCann et al., 2017), precipitation, cloudiness, and moonlight (Beier & McCullough, 1990; Daltry et al., 1998). Hence, daily activity is a trait that can be adapted to a certain extent, to meet a diverse range of environmental pressures (Hayward & Slotow, 2009).

Understanding changes in activity patterns is important because shifts in daily activity may cascade down to other trophic levels (Spoelstra et al., 2015). For instance, predation in forest systems may limit browsing of palatable plants by ungulates in favor of increasing vigilance (Kuijper et al., 2013), providing a window of opportunity for palatable plant species to establish and develop (Ramirez, et al., 2021; Ramirez et al., 2018). This may in turn provide more (high quality) organic material for a more diverse group of macroinvertebrates compared with heavily browsed forests (Allombert et al., 2005; Ramirez, et al., 2021; Wardle et al., 2002). Hence, shifts in animal daily activity patterns can have complex consequences for the entire ecological community.

The impact of anthropogenic factors on daily activity patterns has been studied meticulously (Gaynor et al., 2018), especially for large vertebrates. For example, it is known that large vertebrates strongly adjust their activity to anthropogenic pressure (Gaynor et al., 2018; Spoelstra et al., 2015). For example, coyotes increase nocturnality in response to hunting (Kitchen et al., 2000). Wild boar (Podgórski et al., 2013) and red brocket deer (Di Bitetti et al., 2008) are more nocturnal in areas with higher human presence, while other animals species are found to respond to proximity to roads and logging (Ngoprasert et al., 2017; Ramesh & Downs, 2013). However, no such shifts in activity in response to anthropogenic pressure were found by Di Bitetti et al. (2008) for two related deer species, suggesting species specificity, or involvement of other factors.

Seminal research, however, suggests that animals may shift their activity in the spatial and temporal scales depending on the risk of predation or hunting (Cromsigt et al., 2013; Kronfeld‐Schor & Dayan, 2003). This body of literature is known as Landscape of Fear (Gaynor et al., 2019). For example, foraging may be more efficient in daylight, but also riskier, as predators share the benefit of clear sight. Predation risk may however be reduced by increasing collective vigilance through safety‐in‐numbers, theoretically allowing a more even spread (i.e., a more homogeneous distribution) and higher levels of activity (time spend active) throughout the day (Brown, 1988; Brown & Kotler, 2004; Brown et al., 1999; Gaynor et al., 2019). We know of no studies that consider how daily activity spread and levels throughout the day are affected by population density, even though this may be relevant for activity patterns.

We studied whether the temporal spread and level of daily activity varied with population size in three ungulate species—two of which are social: red deer (*Cervus elaphus*) and wild boar (Sus scrofa), and one of which is a solitary species: roe deer (*Capreolus capreolus*). To do so, we compared diel activity patterns of these species across ten forest sites at the Veluwe, the Netherlands, that widely range in ungulate density. Activity patterns were measured using camera traps. Hunting intensity and food availability were included as covariates because they are known to shift ungulate activity. We tested the predictions that activity spread and level increase with ungulate density only for the social species and not for the solitary species, in agreement with the safety‐in‐numbers theory (Brown, 1988; Brown et al., 1999).

## 2 MATERIALS AND METHODS

### 2.1 Study area

**FIGURE 1:**
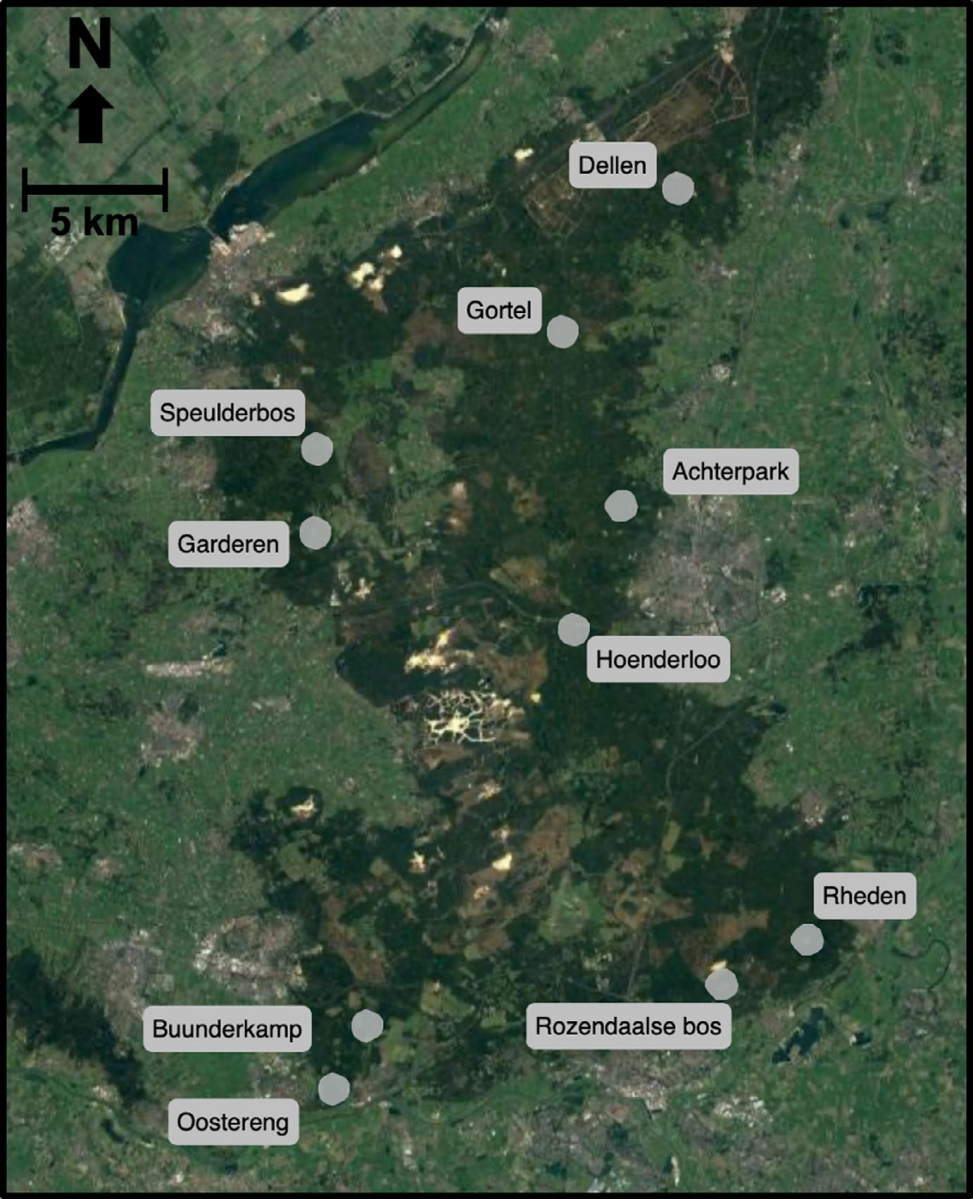
Research sites in the Veluwe, the Netherlands. The gray dots indicate research sites of 1 km^2^, dark green indicates forested areas and light green indicates nonforested areas. Ungulate trap rate per location is provided in Table 1

Fieldwork was conducted during June‐October 2017 at the Veluwe, a 1,200 km^2^ forest‐heathland complex located in the Netherlands (52°11′42″N 5°50′57″E; Figure 1), with an average annual precipitation of 850 mm/year and temperature of 10.5°C. The main soil types are xeric humic podzols and brown earths (Kuiters & Slim, 2002). Assemblages of ungulates vary across the Veluwe and mainly include red deer, roe deer, and wild boar (Ramirez, et al., 2021; Ramirez et al., 2018) with the deer species being crepuscular (Ikeda et al., 2016) and wild boar being a nocturnal species (Podgórski et al., 2013). In 2002, an average ungulate density of 14 individuals/km^2^ was reported for the Veluwe (Kuiters & Slim, 2002) and has increased since. The area is compartmentalized into seven units with contrasting wildlife management regimes and a wide range of ungulate densities. The Veluwe is highly disturbed by human activity, with a high resolution of walking and cycling trails and a series of dense infrastructure scattered across the entire area, leading us to assume an overall equal level of disturbance across our sites.

### 2.2 Sampling design

Across seven management units, we selected ten sites that widely ranged in ungulate density, and also differed in hunting regimes and food availability (Table 1). The minimum distance between sites was 5 km. One contiguous square forest plot of 1 km^2^ was established in each site. Within each plot, 21 random points with at least 100 m interspacing were generated using ArcGIS (ESRI, 2012). At each sampling point, we measured ungulate activity and density with camera traps and food availability with vegetation plots.

**TABLE 1:** Description of the ten study sites at the Veluwe, the Netherlands

Forest site	Management unit	Area size	Sampling effort	Trap rate	Hunting intensity	Food availability
		(km^2^)	(camera days)	(ind m^−1^ day^−1^)	(ind km^−2^ year^−1^)	(ind m^2^)
Oostereng	Zuid Veluwe West	116	516	2.1	0.13	0.47
Achterpark	Veluwe Noord West	242	467	2.3	4.10	0.61
Buunderkamp	Zuid West Veluwe	102	476	5.3	3.83	1.32
Rozendaalse	Zuid Oost Veluwe	197	456	9.0	9.48	0.20
Dellen	Noord Oost Veluwe	130	472	10.2	7.72	0.39
Rheden	Zuid Oost Veluwe	197	400	12.6	9.48	0.51
Speulderbos	Veluwe Noord West	242	446	20.5	4.10	0.56
Garderen	Veluwe Noord West	242	471	21.7	4.10	0.48
Gortel	Ijsselvallei	251	459	65.4	0.01	0.20
Hoenderloo	Midden Veluwe	135	466	101.6	5.84	0.11

Note

Forest site indicates the forest location where cameras were deployed and are ordered by trap rate. Management unit is the administrative division, sampling effort is the total number of days that cameras were deployed, trap rate is a proxy for local ungulate density, hunting intensity is the number of ungulate kills in a given area and time, and food availability indicates the number of tree stems in a given area.

### 2.3 Food availability

To quantify food availability, vegetation plots were paired to the 21 camera points. Vegetation plots were positioned 3 m away from the camera, had a width of 4 m and a variable length to include 50 tree stems of 10–250 cm in height. Food availability was quantified as the density of broadleaved stems per m^2^, since broadleaved species are the preferred food source for our studied species (Ramirez, et al., 2021).

### 2.4 Ungulate density

In each site, three camera traps (Reconyx HyperFire HC500) were deployed for ~21 days at each random point, during June‐October of 2017. Each 21 days, the three camera traps were moved to new points. This procedure was repeated until 21 points had been sampled. This yielded an average of 463 camera trap days per site (Table 1), enough to provide population estimates (Kays et al., 2009).

Camera traps were mounted onto trees at 50 cm height, in steel enclosures with a security cable, facing north, and aimed parallel to the ground. Cameras were set to take bursts of ten images upon each trigger without delay. Vegetation > 50 cm directly in front of the camera was pruned to ensure a free view within the first three meters. Maximum detection distance was estimated at the time of placement as the maximum distance at which a person triggered the camera sensor, allowing to correct for differences in detection distances between camera traps.

Images were grouped into sequences that represented separate animal detections, annotated and stored with the software Agouti (Casaer et al., 2019). The program considered independent visits if the time between triggers took longer than 3 min, this threshold is common for activity studies. For each sequence, species identity, number of individuals, time, and behavior were recorded. As a proxy for ungulate density, we calculated trap rates (ind m¯^1^ day¯^1^), defined as the number of ungulates recorded by the camera traps in proportion to the deployment duration of the camera and the maximum detection distance, as:

$$\text{Trap rate}=\sum_{i=1}^{n}\frac{A}{B\times C}$$

where (*A*) is the total number of ungulates in the image sequences, (B) is the maximal detection distance of the camera in meters, and (*C*) is the sampling effort in days.

### 2.5 Activity patterns

Camera trap captures were grouped by hour of the day to quantify different activity patterns. The spread of activity over the day was calculated using the Shannon index (Shannon, 1948), which is a new approach compared with the use of other activity indicators such as activity level (Rowcliffe et al., 2014) or overlap (Meredith & Ridout, 2014). A higher index value corresponds to a greater spread over the day, that is, more constant activity. It should be noted that the spread itself is not automatically higher when there are more observations. This is because the spread is proportional to the timing of other observations and not to the total number of observations. For instance, a species may have large density difference between sites, but when all individual animals remain active on the exact same moments in time, this will not influence the spread of the activity over the day. Moreover, activity spread differs from activity overlap (Meredith & Ridout, 2014), in that a degree of overlap may indicate whether sites are different from one another, but does not show any directionality of the effect as can be done with an increasing or decreasing activity spread. Activity level was calculated from the circular kernel distribution as described in Rowcliffe et al. (2014) and is defined as the proportion of time spent active by the target species. Variation in day length was modest as all ten sites were surveyed simultaneously during a relatively short period and could therefore be ignored (Vazquez et al., 2019).

### 2.6 Hunting intensity

Hunting intensity was quantified as the take‐off density (ind km^−2^ year^−1^), based on official culling data (http://www.verenigingwildbeheerveluwe.nl/) (Table 1). This essentially corresponds to the density of shots fired rather than the proportion of individuals culled.

### 2.7 Statistical analyses

A principal component analysis (PCA) supplemented with correlation tests was conducted (Perez, 2017) to test for association among fixed factors (ungulate trap rate, food availability, and hunting intensity) and to test for autocorrelation. Linear Models (LM) were used to test for the relationship between activity spread and level with the fixed factors. Analyses were done in R version 3.4.0 using the “vegan” and “activity” packages (Oksanen et al., 2013; R Core Team, 2021; Rowcliffe, 2016).

## 3 RESULTS

The PCA showed large variation among fixed factors. The first principal component explained 40.3% of the variation and was negatively associated with ungulate trap rate. The second explained 35.2% of the variation and was positively associated with hunting intensity and negatively with food availability (Appendix A.1). Based on these results, multicollinearity was rejected and fixed factors were thus included in the analyses.

**FIGURE 2:**
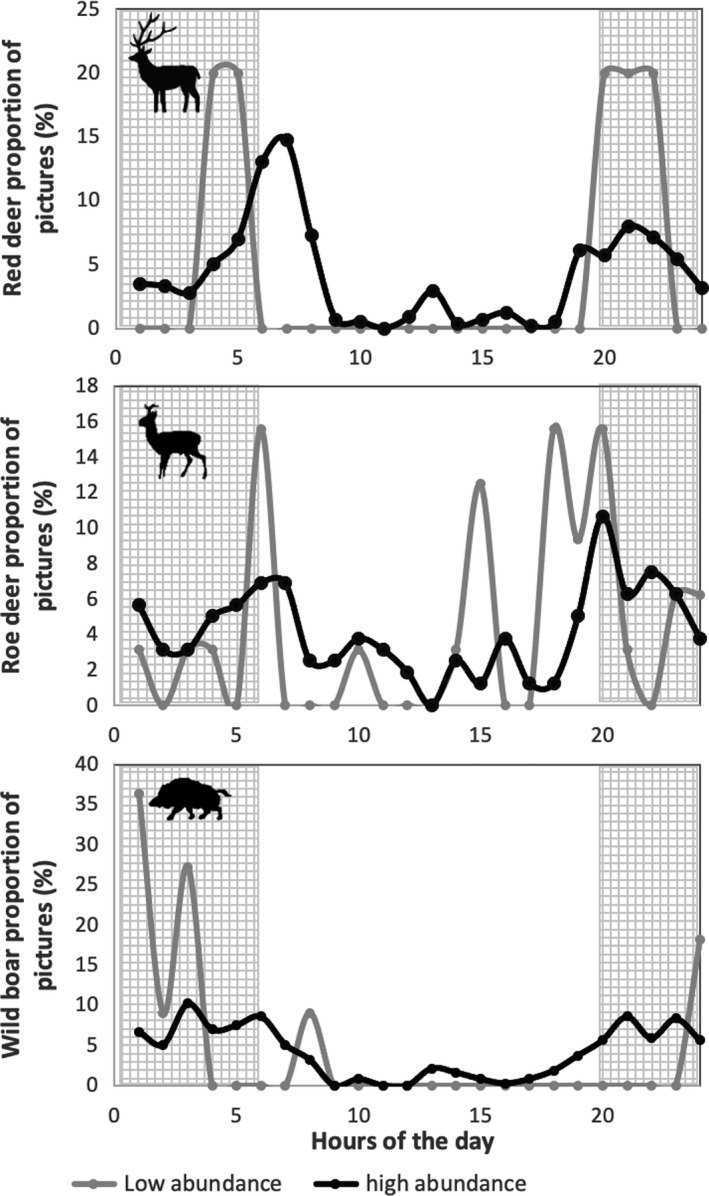
Daily activity patterns for three ungulate species at the Veluwe, the Netherlands, in the two sampled forest sites with the two most contrasting densities: high (black lines) and low (gray lines). (a) Red deer (*Cervus elaphus*) at Garderen and Gortel, (b) roe deer (*Capreolus capreolus*) at Hoenderloo and Buunderkamp, (c) wild boar (*Sus scrofa*) at Buunderkamp and Hoenderloo. See Table 1 for trap rate values. This figure is meant only for illustration purposes

**FIGURE 3:**
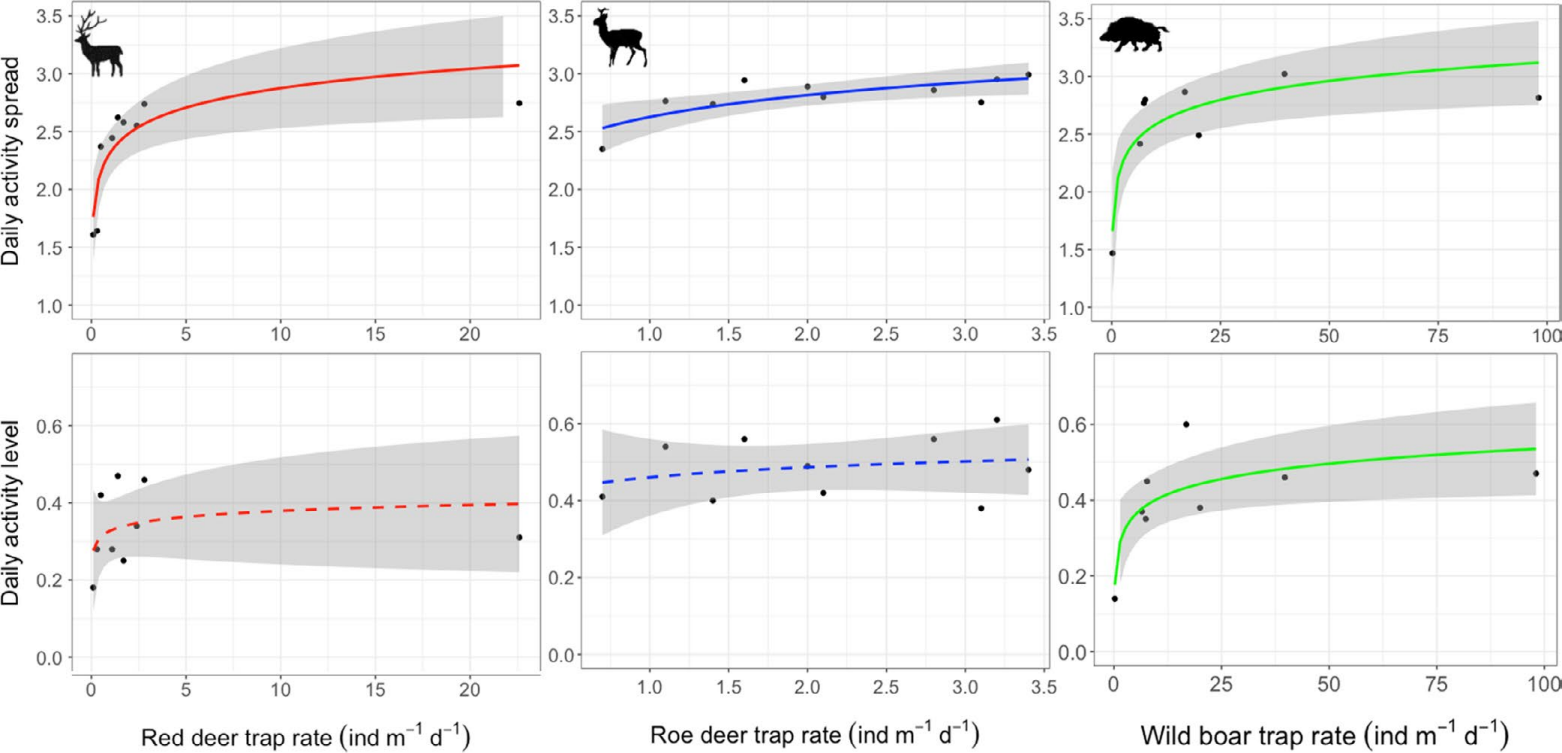
Relations between activity spread and level with trap rate across ten forests at the Veluwe, the Netherlands, for three ungulate species. Lines are Log‐Linear Model fits (Table 2) with confidence intervals. Solid lines represent significant and dashed lines nonsignificant relationships

The spread of daily activity varied widely across the ten sites (ranging from 1.47 to 3.02 H′), as illustrated for low and high abundance sites in Figure 2. For red deer and roe deer, activity peaked in the morning (between 4 and 7 hr) and the evening (between 16 and 21 hr), but these peaks were much more profound at the low‐density site than at the high‐density site (Figure 2a,b). For wild boar, activity peaked during the night (between 23 and 24 hr and between 0 and 4 hr) at sites with low density, but not at sites with high density (between 3–5 hr and between 20–23 hr; Figure 2c). The spread of activity increased with population density in all three species (Table 2; Figure 3). Activity level increased with population density only in wild boar (Table 2; Figure 3). Significance of relationships persisted when the highest and lowest‐density site for red deer and wild boar were removed. Hunting intensity and food availability did not explain any additional variation in the spread or level of activity.

**TABLE 2:** Linear models of the relationship between the spread and level of activity in three ungulate species at the Veluwe, the Netherlands, and ungulate trap rate (ind m^−1^ day^−1^), hunting intensity (ind km^−2^ year^−1^), and food availability (broadleaved stems m^−2^)

Species	*R* ^2^	Sites (*n*)	Coefficients			
			Intercept	Log (trap rate)	Hunting	Food availability
Activity spread						
Red deer	0.82	9	2.32***	0.24**	0.05	0.12
Roe deer	0.69	10	2.63***	0.27**	<0.01	0.17
Wild boar	0.77	8	2.04***	0.24**	<0.01	−0.22
Activity level						
Red deer	0.10	9	0.31*	0.02	0.01	−0.10
Roe deer	0.03	10	0.37**	0.07	0.01	0.01
Wild boar	0.59	8	0.27**	0.06*	0.01	0.07

Note

Numbers given are the coefficient of determination (*R* ^2^), sites (*n*), and absolute regression coefficients of the predictor variables. Significant coefficients are shown with (*).

Significance *p*‐value levels are indicated as: *(0.01 < *x* < 0.05), **(0.001 < *x* < 0.01), ***(*x* < 0.0001).

## 4 DISCUSSION

The safety‐in‐numbers theory predicts that living in higher population densities increases safety for social animals. High‐density populations are thus expected to show a more even spread of their activity and higher activity levels across the day. With camera traps deployed in ten temperate forest sites that ranged in ungulate density, we analyzed the relationships between activity and density in three ungulate species: red deer, roe deer, and wild boar. We found that ungulate activity spread indeed increases with density in all three species, but the relationships were stronger for the social species (red deer and wild boar). Activity level increased only in wild boar. Overall, the results indicate that activity patterns are indeed partly density dependent.

We found that ungulates spread their activity more evenly in sites with higher population density, as predicted. Sites with low deer density had higher activity peaks during twilight and night than sites with high deer density. A similar pattern was observed in nocturnal wild boar, which showed more even activity patterns in sites with high density than in sites with low density. These activity trends could reflect that ungulates feel safer with higher numbers, as theory predicts (Brown, 1988; Brown & Kotler, 2004; Brown et al., 1999), and adjust their activity patterns accordingly (Gaynor et al., 2019). However, a greater spread of activity may also arise in situations of intraspecific competition (Beier & McCullough, 1990). For instance, when dominant individuals push foraging activity of subdominant individuals to suboptimal times of the day (Beier & McCullough, 1990), which may force animals to forage more hours per day (Brown et al., 1999; Mangel & Clark, 1986). Considering this second mechanism, it is remarkable to see that the correlation between spread and density is the weakest for roe deer, which is the only solitary species in this study. This can be explained by the possibility that only one mechanism is at work for roe deer, while both the safety‐in‐numbers and the intraspecific competition may be at work for the other species, thereby increasing the size of the effect. To elucidate whether the safety‐in‐numbers hypothesis or the intraspecific competition for optimal foraging time hypothesis explains ungulate activity patterns, further studies should focus on differences in vigilance or intraspecific dominance in relation to activity. This may be tested by scoring the observed individuals for characteristics such as gender, age and size, and relating these to daily activity.

Results partially supported the prediction that activity levels increase with density because only wild boar activity level increased with population density. This might be because wild boar occur in a wider density range (0.2–98.1 ind m^−1^ day^−1^) than red deer (0.1–22.6 ind m^−1^ day^−1^) and roe deer (0.7–3.4 ind m^−1^ day^−1^) or because wild boar is not a strict browser (Bruinderink et al., 1994). Its diet depends more on fruits, seeds, and roots which might be limited in the Veluwe. Moreover, the safety‐in‐numbers hypothesis implies that animals feel safer to venture out during the day, which may permit them to forage more efficiently due to increased visibility. This in turn may imply that animals require less time to complete their nutrient intake, which could be an explanation for the lack of increase in activity levels for roe deer and red deer.

Animal activity is affected by many environmental variables, making it multifactorial and complex to understand. Research has shown that natural environmental variables such as season, day length, lunar luminosity, and fires play an important role on animal temporal scale (Bennie et al., 2014; Cederlund, 1989; Ikeda et al., 2016). We did not find evidence for this, despite the large gradient in food availability, perhaps because the Veluwe has enough food to support large ungulate populations (0.11–1.32 stems/m^2^).

Human activity, such as hunting, urban development, and land conversion, may even play a more important role in determining activity patterns than natural variables (Gaynor et al., 2018; Kitchen et al., 2000; Ramesh & Downs, 2013; Wang et al., 2015). We did not find evidence for this, despite a large gradient of hunting pressure (0.01–9.48 ind km^−2^ year^−1^) present in our sites. Maybe because hunting in this area is done with high‐power rifles to reduce animal suffering, hence, animals may not perceive hunting cues that they can relate to high risk in both the temporal and spatial scale (Cromsigt et al., 2013). It is important to mention that the studies that do find an effect of hunting on daily activity often compare hunted sites with nonhunted sites (Gaynor et al., 2018) whereas all our sites were hunted to some degree. Although we did not show that the increase in hunting intensity affects activity, this does not preclude that the total absence of hunting will in fact result in altered activity patterns compared to hunted sites.

Population size is generally not accounted for as a factor in daily activity studies (van Doormaal et al., 2015; Nix et al., 2018; Reilly et al., 2017), which may result in false attribution of anthropogenic impacts on a population, while the effects may actually (partly) be the result of the interplay between intraspecific competition and predator avoidance as a function of population size. Several studies reporting decreasing ungulate activity with increasing human activity, also reported decreasing population sizes with increasing human activity (Blake et al., 2013; Di Bitetti et al., 2008; Oberosler et al., 2017). Yet, these studies did not include the lower number of individuals as an explanatory variable. We now show that population density is another important determinant for the way herbivores spread their daily activity. Based on our findings, we suggest to also include population size as a variable in activity studies whenever the study design allows for it.

The relationship between population size and daily activity may not only have an important impact on the fitness of the species itself, but also on other parts of the ecosystem. For example, the diversity of forest recruitment in temperate forest is not only dependent on ungulate density, but also on the time that animals spend foraging. Ungulates sacrifice vigilance and increase browsing time with higher density and therefore release the competition between palatable and less palatable plant species (Ramirez, 2021; Ramirez et al., 2018). The strong‐top down control that ungulates excerpt on the vegetation can also cascade down to other trophic levels, such as invertebrates, rodents (Ramirez, et al., 2021), and song birds (Cardinal et al., 2012) because small invertebrates and vertebrates will not find high‐quality resources.

## 5 CONCLUSION

Our novel parameter—activity spread—accurately captured the variation in ungulate activity as a response to population size, demonstrating its value as an additional tool to analyze animal activity patterns with camera traps. Research results suggest that activity level and spread in ungulates can indeed be density dependent, possibly as a result of increased perception of safety as predicted by the safety‐in‐numbers theory. This suggests that predation risk is counterbalanced by browsing during suboptimal times. We cannot rule out intra‐ and interspecific competition as alternative mechanisms. Further studies are needed to disentangle these alternative drivers. To pinpoint the exact mechanism of how ungulates respond to increasing density, it is necessary to conduct camera trap research that identifies different age groups and gender.

## CONFLICT OF INTEREST

We declare to have no conflict of interests with this publication.

## AUTHOR CONTRIBUTIONS

**Juan Ignacio Ramirez:** Conceptualization (lead); Data curation (lead); Formal analysis (lead); Funding acquisition (lead); Investigation (equal); Methodology (equal); Project administration (equal); Resources (lead); Visualization (lead); Writing‐original draft (equal); Writing‐review & editing (lead). **Joeri A. Zwerts:** Conceptualization (lead); Data curation (lead); Formal analysis (lead); Funding acquisition (equal); Investigation (equal); Methodology (equal); Project administration (equal); Writing‐original draft (equal); Writing‐review & editing (supporting). **Marijke van** **Kuijk:** Conceptualization (supporting); Funding acquisition (lead); Investigation (supporting); Methodology (supporting); Project administration (supporting); Supervision (lead); Writing‐original draft (supporting); Writing‐review & editing (supporting). **Palma** **Iacobelli:** Data curation (supporting); Formal analysis (supporting); Investigation (supporting); Writing‐original draft (supporting). **Xuqing Li:** Data curation (supporting); Formal analysis (supporting); Investigation (supporting); Writing‐original draft (supporting). **Natalie** **Herdoiza:** Conceptualization (supporting); Investigation (supporting); Methodology (supporting); Writing‐original draft (supporting). **Patrick A. Jansen:** Conceptualization (supporting); Formal analysis (supporting); Funding acquisition (lead); Investigation (supporting); Methodology (supporting); Supervision (lead); Writing‐original draft (supporting); Writing‐review & editing (supporting).

## Figures and Tables

**FIGURE A1:**
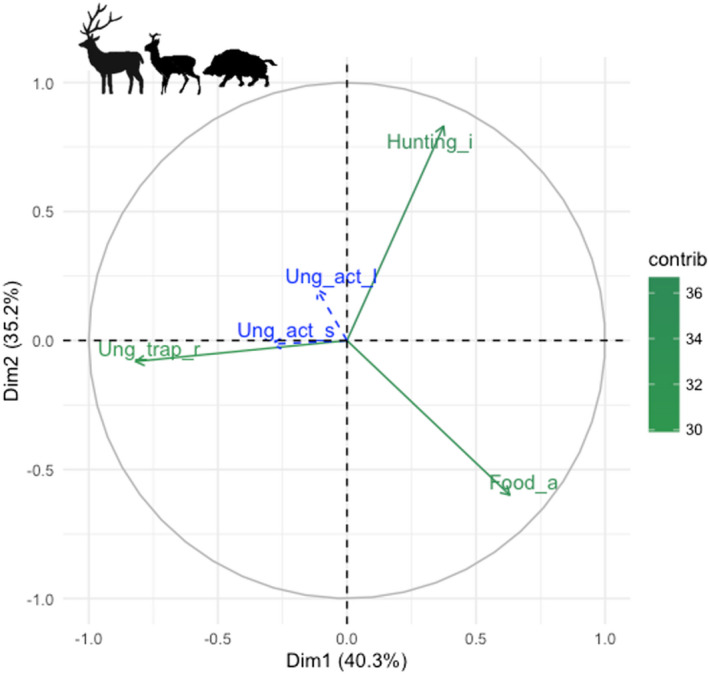
Principal component analysis (PCA) for ungulate landscape trap rate, food availability, and hunting intensity across the study sites at the Veluwe, the Netherlands. The analysis is based on 212 sampling points scattered across ten research sites. The length of the arrow is proportional to its importance and the length combined with the angle between two arrows reflects the magnitude of the correlation between variables. Variable were coded in green as: Ung_trap_r = Ungulate trap rate, Food_a = food availability, and Hunting_i = hunting intensity. Superposed variable in blue was coded as: Ung_act_s = ungulate activity spread and Ung_act_l = ungulate activity level. Correlation tests confirm that none of the fixed factors are correlated (Correlation tests: Ung_trap_r − Hunting_i = −0.14, Ung_trap_r − Food_a = −0.18, Hunting_i − Food_a = −0.06)

## Data Availability

Underlying data are available on the Dryad digital repository (https://doi.org/10.5061/dryad.m63xsj41x).

## APPENDIX A

**TABLE A1:** Total captures of red deer, roe deer and wild boar by our camera trap network deployed across the ten forest sites in the Veluwe

Forest site	Red deer	Roe deer	Wild boar	Total
Oostereng	0	127	0	127
Achterpark	11	87	1	99
Buunderkamp	96	159	11	266
Rozendaalse	43	44	188	275
Dellen	40	66	229	335
Rheden	79	119	173	371
Speulderbos	18	117	366	501
Garderen	5	80	438	523
Gortel	576	96	989	1,661
Hoenderloo	116	32	371	519
